# Systematic bioprospection for cellulolytic actinomycetes in the Chihuahuan Desert: isolation and enzymatic profiling

**DOI:** 10.7717/peerj.16119

**Published:** 2023-09-28

**Authors:** Janneth Escudero-Agudelo, Juan Martínez-Villalobos, Hector Arocha-Garza, Luis Jesús Galán-Wong, Hamlet Avilés-Arnaut, Susana De la Torre-Zavala

**Affiliations:** Universidad Autónoma de Nuevo León, Facultad de Ciencias Biológicas, Instituto de Biotecnología, San Nicolás de los Garza, Nuevo León, México

**Keywords:** Celullases, Actinomycetes, Cuatro cienegas basin, *Streptomyces*

## Abstract

The quest for microbial cellulases has intensified as a response to global challenges in biofuel production. The efficient deconstruction of lignocellulosic biomass holds promise for generating valuable products in various industries such as food, textile, and detergents. This article presents a systematic bioprospection aimed at isolating actinomycetes with exceptional cellulose deconstruction capabilities. Our methodology explored the biodiverse oligotrophic region of Cuatro Cienegas, Coahuila, within the Chihuahuan Desert. Among the evaluated actinomycetes collection, 78% exhibited cellulolytic activity. Through a meticulous screening process based on enzymatic index evaluation, we identified a highly cellulolytic *Streptomyces* strain for further investigation. Submerged fermentation of this strain revealed an endoglucanase enzymatic activity of 149 U/mg. Genomic analysis of strain *Streptomyces* sp. STCH565-A revealed unique configurations of carbohydrate-active enzyme (CAZyme) genes, underscoring its potential for lignocellulosic bioconversion applications. These findings not only highlight the significance of the Chihuahuan Desert as a rich source of cellulolytic microorganisms but also offer insights into the systematic exploration and selection of high-performing cellulolytic microorganisms for application in diverse environmental contexts. In conclusion, our bioprospecting study lays a foundation for harnessing the cellulolytic potential of actinomycetes from the Chihuahuan Desert, with implications for advancing cellulose deconstruction processes in various industries. The findings can serve as a blueprint for future bioprospecting efforts in different regions, facilitating the targeted discovery of microorganisms with exceptional cellulosic deconstruction capabilities.

## Introduction

The quest for alternative energy sources to mitigate climate change has driven the search for microbial enzymes that can sustainably use plant cellulose, the most abundant type of biomass, but also a recalcitrant resource of biological energy (van Vuuren et al., 2017). Achieving inexpensive and environmentally sustainable complete deconstruction of lignocellulose is still a pending task. To address this, researchers are tackling the search for new sources of cellulases by (i) developing cellulolytic consortia (Zhang & Dong, 2022), (ii) screening microorganisms that can survive and function in extreme conditions (Barzkar & Sohail, 2020; Champreda et al., 2019; Elleuche et al., 2014; Sysoev et al., 2021; Verma, 2021; Wu et al., 2018) and (iii) synthetically enhancing cellulases by developing chimeric enzymes (Banerjee et al., 2016; Dadwal, Sharma & Satyanarayana, 2020; Guazzaroni, Silva-Rocha & Ward, 2015). The discovery of new genetic resources and diverse functionality in the enzymatic activity will result in cost-effective, low-energy, and environmentally friendly biofuel producing processes.

Extreme environments have shaped biodiversity driven by harsh physicochemical conditions displayed in a myriad of adaptations in microorganisms of high biotechnological value, including microbial enzymes for a plethora of applications (Manni & Filali-Maltouf, 2022; Pascoal, Magalhaes & Costa, 2020; Sysoev et al., 2021; Tatta et al., 2022; Uma et al., 2020; Wani et al., 2022a). Bacterial enzymes possess key features more advantageous than fungal enzymes such as higher growth rates, genetic versatility (Menendez, Garcia-Fraile & Rivas, 2015) and more specific, efficient catalytic activity in harsh conditions; moreover, bacteria produce multienzyme complexes with greater functionality and specificity in broader conditions (Balla et al., 2022; Castiglia et al., 2016; Maki, Leung & Qin, 2009). Cellulases from actinomycetes associated to insects and other extreme and marine prokaryotes have exhibited high specific activity, thermostability, and other important biochemical properties and hence can contend well with the enzymes from terrestrial sources (Behera et al., 2017; Elframawy et al., 2022; Fathallh Eida et al., 2012; Gong et al., 2017; Gong et al., 2020; Goodfellow et al., 2018; Lewin et al., 2016; Saini et al., 2015; Walia et al., 2017; Xie & Pathom-Aree, 2021).

The Cuatro Ciénegas Basin (CCB) in Northern Mexico is an extreme, oligotrophic environment, and a hotspot of biodiversity thriving in endangered oasis in the Chihuahuan desert (Souza et al., 2012b). Here, the proportions of nitrogen and phosphorus (N:P) are extremely skewed, given the great limitation of phosphorus (P) (157:1) (Elser et al., 2005) or very low nitrogen (N) (1.8:1) (Souza et al., 2006) and sulfur and magnesium (Mg) that replicate marine osmolarity, while being low in NaCl (De Anda et al., 2017; Rebollar et al., 2012; Souza et al., 2018; Wolaver et al., 2012). The Churince System is the most unusual hydrological system within the CCB due to its higher altitude within the valley (around 30 m above most of the basin); the system depends on mostly deep ancient water with a magmatic influence (Wolaver et al., 2012) and a calcium soil matrix (Elser et al., 2005). This peculiar environment had favored a great diversity of microorganisms that have thrived in conditions of scarcity and isolation, creating a so-called “lost world” (Souza et al., 2018), including diverse actinobacteria, proposed to be endemic to CCB ponds (Arocha-Garza et al., 2017) and metabolically unique (Gonzalez-Salazar et al., 2023) have been previously described.

Despite accumulating evidence of catalytic novelty in non-culturable independent studies (Georgiadou et al., 2021; Manni & Filali-Maltouf, 2022; Schaller et al., 2022; Sysoev et al., 2021; Wani et al., 2022a; Wani et al., 2022b), and reports from isolated highly valuable microbial collections from unexplored sites (Gonzalez-Salazar et al., 2023; Swiecimska, Golinska & Goodfellow, 2022), there is still a need for efficient and detailed systematic bioprospecting studies for cellulolytic actinomycetes. Work describing scrutinization criteria and the rationale behind the selective methodology of microbial collections could enhance bioprospection efforts in underexplored biomes that will effectively filter “gifted microbes” harboring more valuable enzymes and other natural products. This work aimed to develop a systematic detailed bioprospecting exploration of actinomycetes isolated from the CCB oligotrophic ponds, with high potential of enzymatic deconstruction of cellulose.

## Materials & Methods

### Sampling site and actinomycetes collection

**Figure 1:**
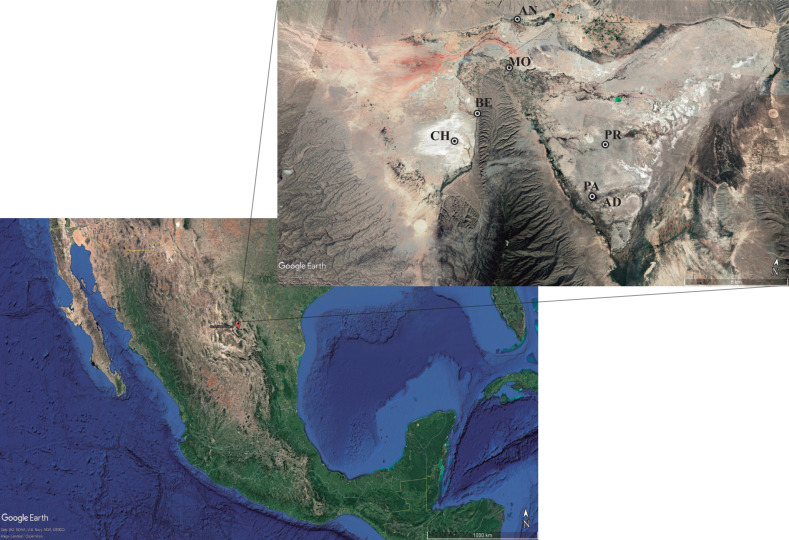
Sampling sites for actinomycetes isolation. The hydrological system locates the Cuatro Ciénégas Basin (CCB) in the Chihuahua desert, México. Localization: (CH) Churince system (26‘50′58″N 102‘09′12″W); (BE) Becerra (26‘52′42″N 102‘08′17″W); (MO) Mojarral (26‘55′22″N 102‘07′06″W); (AN) Anteojo (26‘58′08.95″N 102‘07′13.84″O); (PR) Pozas Rojas (26‘33′00″N 102‘27′32″W); (AD) Archaean Domes (26‘49′41.70″N 102‘01′28.74′’O); (PA) Pozas Azules (26‘55′22″N 102‘07′12″W). Map data: ©2022 Google LLC, Maxar Technologies.

The complete actinomycetes collection consisted of 196 isolates from seven ponds in the Cuatro Ciénegas Basin (Fig. 1). Isolates belong to the Microbiological Collection of Instituto de Biotecnología-UANL and the data is described in (Table S1). Nearly 90% of isolates had been previously reported (Arocha-Garza et al., 2017; Arocha-Garza, 2018). Access and permission to Natural Protected Area was given by SEMARNAT in document id.no. SGPA/DGVS/01644/19. Only 20 actinomycetes from the collection are newly reported in this work. Briefly, 5 g of sediment from the pond were retrieved with approximately 35 mL of the water column from the pond. Samples were not refrigerated, instead, samples were immediately (2–6 h after sampling) transferred by plating 200 µL of the sediment-water suspension to isolation media (Table S1). Plates were incubated at 28°–30 °C for 7–10 days or until single colonies were able to be picked and transferred to a clean plate for secondary isolation.

### Characterization of actinomycetes isolates collection

The Cuatro Ciénegas actinomycetes collection was first characterized microscopically, confirming filamentous prokaryotic gram-positive structures, and macroscopically by colony morphology in ISP media (Li et al., 2016). Long-term storage of biological collections was set up in 50% glycerol and preserved at −20 °C.

### Screening and selection of highly cellulolytic actinomycetes

**Figure 2:**
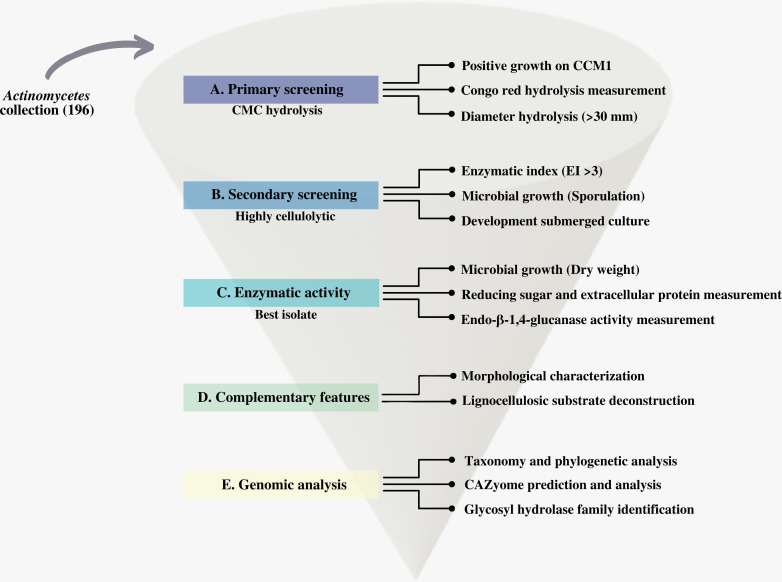
Scheme of the systematic bioprospecting for the retrieval and characterization of highly cellulolytic actinomycetes in Cuatro Ciénegas Basin (CCB), México.

We first screened our CCB actinomycetes collection for cellulolytic isolates. We designed and applied a systematic process that we summarize in Fig. 2.

#### Primary screening of cellulolytic activity

A primary screening of the cellulolytic potential of axenic cultures of actinomycetes was performed by the petri plates assay method. Actinomycete collection strains were inoculated on carboxymethylcellulose medium-CCM1-(Carboxymethylcellulose sodium salt (CMC) 10 g/L, K_2_HPO_4_ 2 g/L, (NH_4_)_2_ SO_4_ 0.35 g/L, urea 0.0748 g/L, agar 15 g/L, KENT^®^ Marine Reef Salt 25 g/L, a pH 7.0 ± 0.1) as follows: (i) an agar plug of 0.5 cm diameter from actively growing actinomycete–sporulated ISP2 culture was transferred to the center of a CCM1 agar plate. (ii) plates were incubated at 30 °C for 10 days; (iii) growth was considered positive when active vegetative growth was >2 mm of length around the agar-plug inoculation area; (iv) Congo-red test was used to measure the diameter of the hydrolysis zone after ten days of culture as an indicator for cellulose degradation (Gupta, Samant & Sahu, 2012). The experiment was performed using three replicates for each strain. The average of three experiments was calculated with the corresponding standard error of the mean. Isolates displaying growth on CMC1 (>2 mm) and a hydrolysis zone revealed by Congo red -(>30 mm diameter), were selected for secondary screening evaluation. Strains displaying growth <2 mm on CCM1, or a cellulose degradation zone <30 mm diameter, were discarded. In addition to the previously described biological collection, two positive controls were included: a strain of *Streptomyces coelicolor*, previously described as cellulose degrading and a *Trichoderma harzianum* strain from Instituto de Biotecnología Microbial Collection. Negative controls were also included: an *Escherichia coli* strain, and non-inoculated plate was used as negative control.

#### Secondary screening of cellulolytic actinomycetes by determination of Enzymatic Index

Twelve isolated actinomycete strains with larger hydrolysis zones (>30 mm diameter) were selected for secondary screening. These microorganisms were incubated for seven days at 27 °C on CCM1 medium previously described. The measurement of the hydrolysis halo and of the colony was performed with the ImageJ software (version 1.2) analysis program by calibrating the program with the measurement scale present in each acquired image. The enzyme index (EI) (da Cruz Ramos et al., 2016; Florencio, Couri & Farinas, 2012; de Paula et al., 2016; Soy, Nigam & Sharma, 2019), of each strain was calculated as: $EI= \frac{Hydrolysis~zone~diameter}{Colony~diameter} $

#### Statistical analysis of Enzymatic Index determination experiment

The presented experiment was performed using three replicates for each strain. The average of three replicates was calculated and the standard error of the mean (SEM). The statistical adequacy of the model was determined through analysis of variance (ANOVA) and Tukey test, with 95% confidence performed Minitab Statistical software (Version 17). The visualization of results was graphed using GraphPad Prism software (version 9.2.0).

### Measurement of cellulolytic activity

#### Crude cellulolytic fraction

A pre-inoculum of the strain displaying the highest enzymatic index was prepared to inoculate a submerged fermentation. Vegetative growth shown in not-sporulated colonies was removed from the Petri dish and used to inoculate liquid CCM2 (described below) supplement with glucose to obtain 0.1 optical density at 600 nm (A_600nm_). The culture medium (CCM2) for this experiment consists of [per liter]: CMC 5 g, glucose 1 g, K_2_HPO_4_ 2 g, (NH_4_)_2_SO_4_ 0.35 g, urea 0.0748 g, Reef salt mix Kent^®^ 5 g, at pH 7.0 ± 0.1. The experiment was carried out in triplicate in 500 mL Erlenmeyer flask containing 200 ml of medium. Flasks were incubated at 37 °C for 20 days in a shaker incubator at 150 rpm. A volume of 2.0 mL was withdrawn each day from the fermentation flask and centrifuged before use at 12,000 rpm/10 min to separate the crude enzyme (CE) of the other cellular *detritus* and stored at −20 °C.

#### Endo-β-1,4-Glucanase Activity determination

The clear supernatant broth was collected aseptically and filter-sterilized for further determination of enzymatic activity (crude cellulolytic fraction). The endo-β-1,4-glucanase volumetric enzymatic activity (EndG) was quantified using 2% (w/v) CMC solution as a substrate in 0.05 M sodium citrate buffer pH 4.8 (Ghose, 1987) using triplicates. The amount of reducing sugars released after 30 min at 50 °C was determined by the 3,5 -Dinitrosalicyclic acid (DNS) method (Miller, 1959) and the A_540nm_ was used for glucose standard curve. One unit of EndG was defined in terms of International Unit (IU) as the amount of enzyme that releases 1 µmol of glucose per min under the conditions. Specific activity of the CE was determined as U/mg of protein.

#### Extracellular protein

The total extracellular protein content was measured in triplicates using the protein reagent (Sigma-Aldrich), consistent with the Bradford method (Bradford, 1976), bovine serum albumin was used as the standard and values were measured at A_595nm_ (microprotein assay).

#### Microbial growth

The dry weight (solid content) of bacterial cells in suspension was obtained by drying an established volume in an oven at 105 °C to constant weight. In this study, 1000 µL of sample was obtained every 24 h during the 20 days of evaluation (the small pellets produced in the submerged culture, did not need disaggregation elimination procedure, and possible pipetting inconsistencies, were controlled using a trimmed micropipette tip, increasing the diameter available for sampling). The sample was centrifuged, and the pellet obtained was dried at 105 °C in a conventional drying oven. Subsequently, and to eliminate any remaining moisture in the microtube, it was dried to constant weight at 40 °C. Dried samples in the microtubes were weighed on an analytical balance. Prior to the experiment, the microtubes were weighed empty the value of the ’weight with sample - weight of the empty tube’ and to calculate the biomass of the microorganism during the fermentation test. The results were analyzed from the triplicate of the experiment and were calculated as weight loss or gain in mg/L.

#### Assay for demonstration of deconstruction of diverse lignocellulose substrates

Isolated actinomycete strain with highest enzymatic index was evaluated on diverse lignocellulosic substrates, including Avicel^®^, xylan and lignin. For this, plate Petri was used. The culture was incubated at 27 °C for seven days. The culture media used in this experiment are described below [per liter]: LAM medium (Lignin alkali) 5 g/L, (NH_4_)_2_ SO_4_ 1.4 g/L, K_2_HPO_4_ 2 g/L, urea 0.3 g/L, agar 20 g/L, KENT^®^ Marine Reef Salt 15 g/L; XYM medium (xylan hydrolyzed from corncob) 10 g/L, K_2_HPO_4_ 2 g/L, (NH_4_)_2_ SO_4_ 1.4 g/L, urea 0.3 g/L, agar 20 g/L, KENT^®^ Marine Reef Salt 15 g/L; ACM medium (Avicel^®^ PH-101) 10 g/L, K_2_HPO_4_ 2 g/L, (NH_4_)_2_ SO_4_ 1.4 g/L, urea 0.3 g/L, agar 20 g/L, KENT^®^ Marine Reef Salt 15 g/L. *Trichoderma harzanium* and *Escherichia coli* were included as positive and negative controls for the deconstruction of the lignocellulosic substrates experiments.

### Morphological characterization

According to the proposed bioprospecting route (Fig. 2D) the final selected strain was characterized by means of morphological features of vegetative and sporulated growth in International *Streptomyces* Project culture media (Shirling & Gottlieb, 1966). Macro-morphological, micro-morphological and physiological including Gram stain and fresh observation features were studied. Characterization of the strain was made using the standard ISP medium. The color of aerial mycelium, substrate mycelium and soluble pigment was observed with the naked eye. The physiological test included melanin synthesis by diffusible pigments over culture plate and carbon utilization. Axenic cultures and microbial growth were corroborated in each step.

### Whole genome sequencing

Genomic DNA of *Streptomyces* sp. STCH565-A was obtained using a modified phenol/chloroform method that yielded the best quality DNA for our isolate, following the methodology proposed by Arocha-Garza et al. (2017). Total DNA of the sample was sent to CINVESTAV-LANGEBIO, Irapuato, Mexico, for shotgun whole genome sequencing using Illumina Mi-Seq 2*300 platform according to the manufacturer’s protocol (Illumina Inc., USA).

### *De novo* assembly, gene prediction and genome annotation

Sequence adaptors and low-quality reads were trimmed with Trim Galore v0.6.6 (Krueger, 2012) and BBDuk (Bushnell, Rood & Singer, 2017), the resulting reads were assembled into contigs using Unicycler v2.12.0 (Wick et al., 2017). The contigs were filtered for minimum size (300 bp) and minimum read support (Blin et al., 2019). To evaluate the integrity of the assembled genome, the longest contigs were compared using BLASTn v2.12.0+ against bacteria genomes from the GenBank database. No contamination was observed with this method.

To annotate the contigs, the nucleotide sequence was uploaded to the PATRIC RASTtk-enabled Genome Annotation Service v.3.6.12 (Brettin et al., 2015) online server (https://patricbrc.org/). This service was subjected to parameters focused on bacteria such as the genus *Streptomyces* (Taxonomy ID 1883) with genetic code 11 (Archaea & most bacteria) to find most of the protein-coding genes as well as tRNAs and rRNAs. The whole genome project was deposited at GenBank under the accession PRJNA845783. The possible identification of plasmid was conducted using RFPlasmid (van der Graaf-van Bloois, Wagenaar & Zomer, 2021) and PlasForest (Pradier et al., 2021). The biosynthetic novelty index (BiNI) (Gonzalez-Salazar et al., 2023) was calculated for *Streptomyces* sp. STCH565-A.

### Genomic analysis

#### Carbohydrate-active enzyme exploration

The putative genes encoding Carbohydrate-active enzyme (CAZy) were genome-mined using the dbCAN2 meta server (Zhang et al., 2018) and handled with the R package Tidyverse (Wickham et al., 2019) to identify glycoside hydrolases (GH) (Drula et al., 2022; Lombard et al., 2014), as well as the auxiliary activities and substrate-binding domains corresponding to the classification of the CAZy database (http://www.cazy.org). Considering the results of dbCAN, GenBank annotation and sequence alignment, a potential activity was assigned to each CAZyme.

#### Phylogenomic analysis

Taxonomy assignment was performed using the Genome Taxonomy Database (GTDB-Tk) workflow (Chaumeil et al., 2019); the genome was analyzed to predict genes as phylogenetic markers using Prodigal (gene calling) from a bacterial 120-marker set based on TIGRFAM and Pfam databases. The result was aligned using hmmalign to determine the closest domain and the output markers were concatenated to determine their most likely classification by placing the genome into a reference tree within 45,555 available genomes from GTDB (Parks & Hugenholtz, 2022). The resulting tree was first visualized in Dendroscope to retrieve species within the *Streptomyces* genus (Huson et al., 2007) and it was finally adapted using the Interactive Tree of Life (iTOL) v4 (Letunic & Bork, 2019) by collapsing clades at 2.5 average branch length. All the final figures were adapted in Inkscape (Yuan et al., 2016).

## Results

### Screening and selection of highly cellulolytic bacteria

This study performs a systematic bioprospection of the Cuatro Ciénegas Basin (CCB) for the isolation and evaluation of actinomycetes retrieved from an extreme environment with demonstrable cellulolytic capacity (Fig. 2). The study explored an extensive and diverse actinomycete collection from the CCB for the characterization of β-1,4-glucanase–producing actinomycete strains. Seven ponds in CCB were sampled: Pozas Rojas (PR), Pozas Azules (PA), Mojarral (MO), Becerra (BE), Archaean Domes (AD), Anteojo (AN), and Intermediate Lagoon in the Churince System (CH) (Fig. 1) and 196 morphologically distinct actinomycete isolates were subjected to primary screening using CCM1 media (Fig. 2A).

In this primary screening, 156 of the 196 evaluated strains were considered cellulolytic (78% of total actinomycetes strains) (Table S1), because they had the ability to grow on CCM1 media. Two large groups of cellulolytic microorganisms, those whose hydrolysis halo size was <30 mm in diameter, were considered to have less cellulose degradation. Actinomycetes whose halo was >30 mm in diameter were identified with the greatest potential to degrade cellulose under the conditions of this study (Fig. 2B). According to our study, the positive control (*S. coelicolor*) strain obtained a hydrolysis halo <30 mm in diameter.

**Figure 3:**
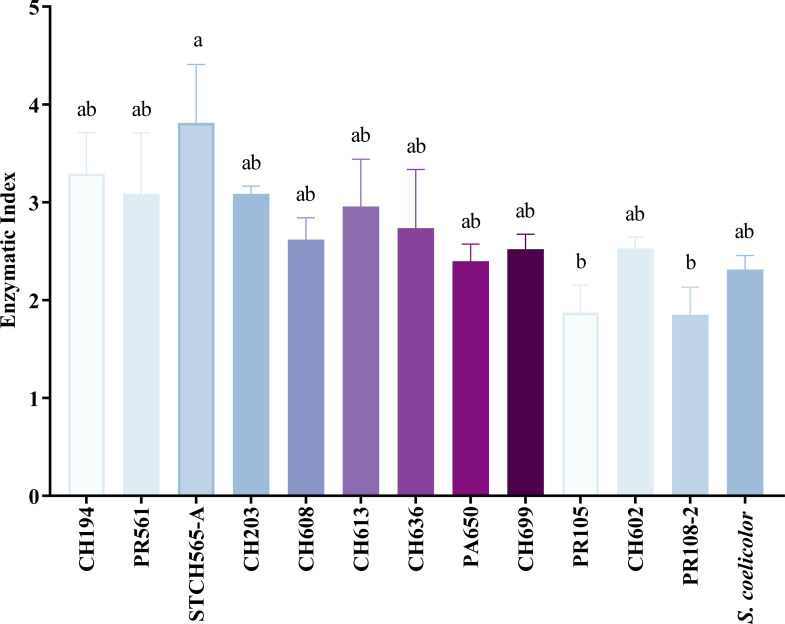
CCB isolates cellulolytic enzymatic index (EI). EI calculated after 7 days of evaluation on CCM1 agar medium. All values represent the mean ± standard error of three independent experiments. Significant differences were determined using analysis of variance (ANOVA) followed by Tukey’s test (*p* < 0.05).

Of the 156 cellulolytic isolates, 12 showed a significantly large diameter of hydrolysis zone values (>30 mm) and were selected for further evaluation in plate assay method in a secondary screening (Fig. 2B). After calculating enzymatic index (EI), (da Cruz Ramos et al., 2016; Florencio, Couri & Farinas, 2012; de Paula et al., 2016; Soy, Nigam & Sharma, 2019), our results showed two major groups in our collection, one with an enzyme index above two and one with values below two as shown in Fig. 3. Statistical analysis showed significant difference of isolates with EI values below two (*p*-value = 0.043), *i.e.,* group *b* (PR105, PR108-2). However, the isolated STCH565-A stands out among other isolates not only for its highest enzymatic index (EI = 3.8) but also for the larger halo diameter zone (Fig. 3).

### Measurement of enzyme activity

**Figure 4:**
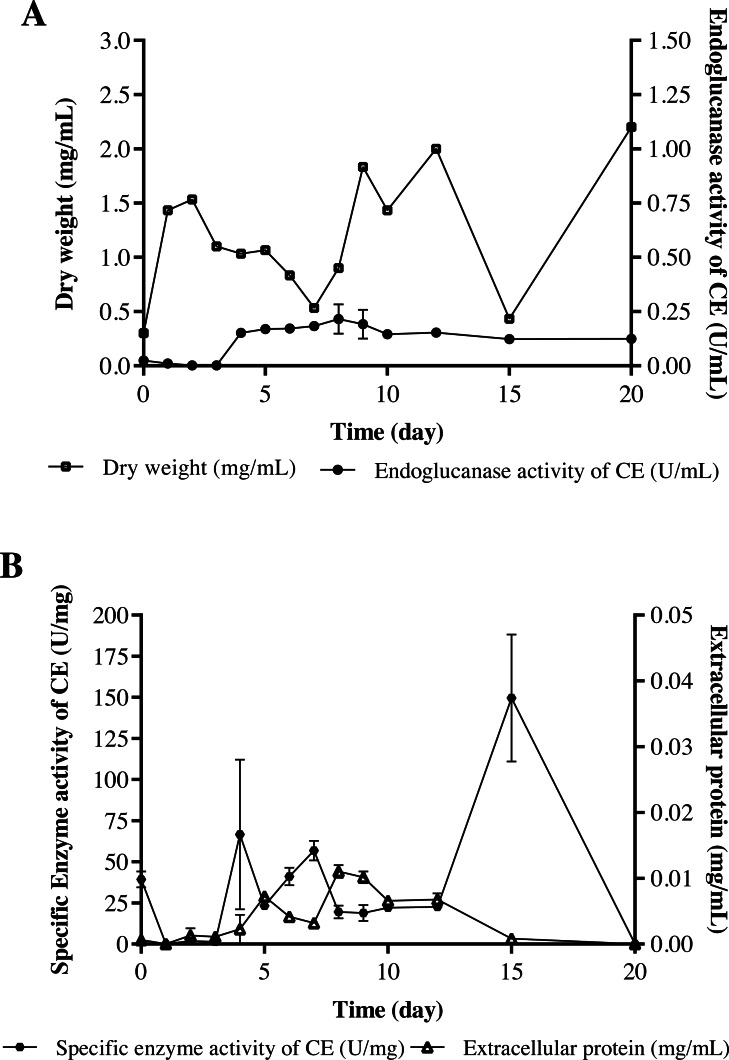
Cellulolytic activity of crude extract of the microorganism STCH565-A in CMC minimal medium during 20 days of evaluation. All values represent the mean ± standard error of three independent experiments. (A) Volumetric enzymatic activity and reducing sugars released by crude extract. (B) Specific activity of crude extract and extracellular protein.

The strain *Streptomyces* sp. STCH565-A was finally selected to continue with the measurement of endoglucanase activity (Fig. 2C). In a submerged culture, biomass presented several development events. At the beginning, dry weight increases probably due to the immediate consumption of glucose, as it is a less complex carbon source and implies less energy expenditure for the microorganism, therefore, a preference for it is assumed. After a brief fall, biomass stabilizes, followed by a second fall before increasing and finally a fall before a last increase, which coincides with the sporulation of the microorganism. Endo- β-1,4-glucanase activity (in terms of volumetric activity) values are reported from day 4 of culture, coinciding with the consumption of glucose available in the medium; these values ranged from 0.15 U/mL to 0.27 U/mL (Fig. 4A). Regarding the enzymatic activity, three important moments to consider were reported, with values ranging from 56 U/mg to 149 U/mg (Fig. 4B).

### Morphological characterization

Actinomycete strain STCH565-A formed a well-developed aerial mycelium with good sporulation on ISP solid medium. The morphological characteristics of STCH565-A growth, substrate mycelium color, aerial mycelium color, and pigment are presented (Table 1). Noticeably, STCH565-A isolate displayed melanin pigment in ISP6 and ISP7 agar medium (Table 1). Sucrose was the best carbon source exhibited maximum growth, as well as STCH565-A exhibited growth on arabinose, xylose, mannitol, fructose, Rhamnose and effectively on cellulose. While on inositol STCH565-A did not show growth (Table 1).

**Figure 5:**
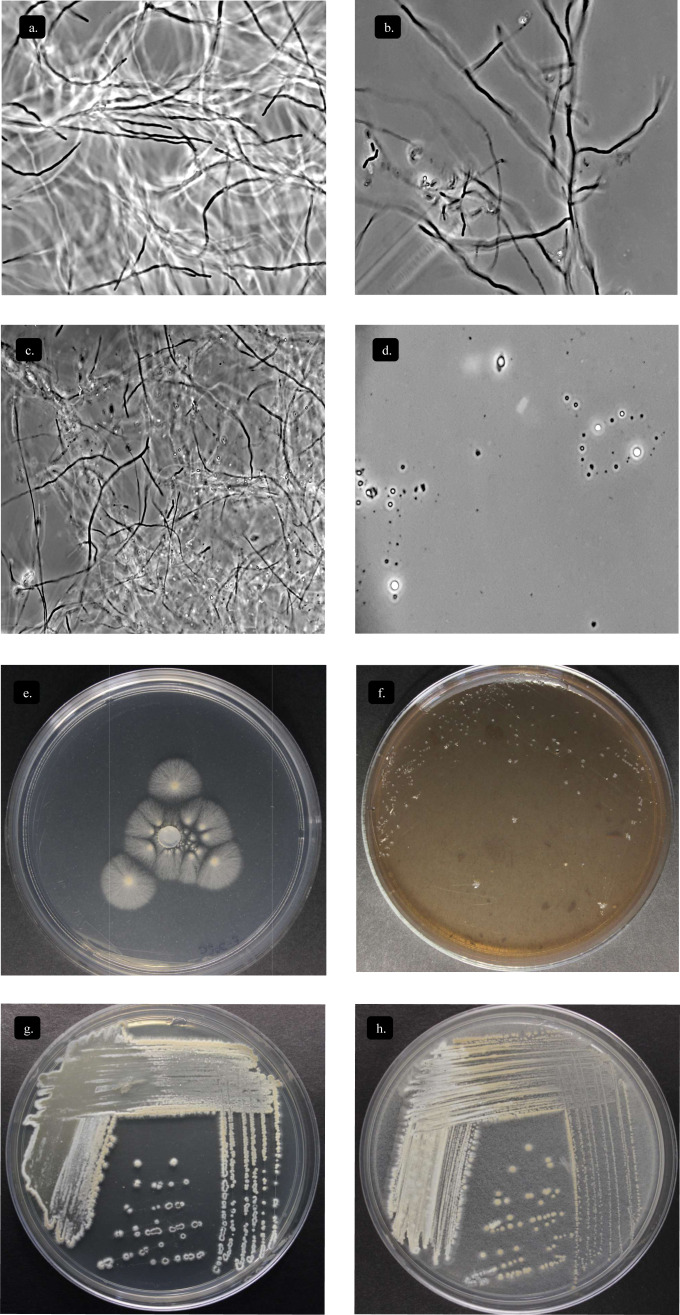
Characterization *Streptomyces* sp. STCH565-A. Microbial growth by phase contrast microscopy (100x) in submerged cellulose medium. (A) Day 1 (B) Day 7 (C) Day 12 (D) Day 15. Growth on other sole-carbon sources from lignocellulose. (E) Cellulose (F) Lignin (G) Xylan (H) Avicel^®^.

Below there is a general description of the observations of strain STCH565-A in liquid culture medium in Fig. 5A. Three stages of development can be identified in the microorganism at the macroscopic level: the first corresponds to vegetative growth on the substrate with yellow biomass, followed by the beginning of sporulation which is observed in white and finally a stage of aerial growth (sporulation) lilac white, lilac, or light violet depending on the color scale used (Table 1).

**Table 1:** Characteristics of *Streptomyces* sp. STCH565.A grown in different media.

*Characters studied*	*Features*	
**Morphological characteristics**	**Substrate mycelium**	**Aerial mycelium**
Tryptone-yeast extract broth (ISP1)	Yellow	White
Yeast malt extract agar (ISP2)	Light yellow	Lilac
Oatmeal agar (ISP3)	Transparent Ivory	Ivory
Inorganic salt agar (ISP4)	Light yellow	Yellow orange
Glycerol asparagine agar (ISP5)	Yellow	Cream-white
**Physiological characteristics**	**Diffusible pigments**	
Peptone yeast extract agar (ISP6)	+	
Tyrosine agar (ISP7)	+	
**Carbon utilization test**	*Colony Growth	
Arabinose	+	
Sucrose	++	
Xylose	+	
Inositol	-	
Mannitol	+/-	
Fructose	+	
Rhamnose	+	
Cellulose	+	

**Notes.**

Assay were carried out as mentioned in Shirling and Gottlieb (1966).

* Strongly positive utilization (++) when growth on tested carbon in basal medium is equal to or greater than growth on basal medium plus glucose.

Positive utilization (+) when growth on tested carbon is significantly better than on basal medium without carbon, but somewhat less than on glucose.

Utilization doubtful (+/-) when growth on tested carbon is only slightly better than on the basal medium without carbon and significantly less than with glucose.

Utilization negative (-) when growth is like or less than growth on basal medium without carbon. (Always record utilization as negative if growth is not better than no-carbon control.

### Deconstruction of lignocellulosic substrates

*Streptomyces* STCH565-A was subjected to enzyme screening using three different lignocellulose media (Table 1). When Avicel^®^ and xylan substrate were used, growth was observed, while on lignin substrate the microorganism *Streptomyces* STCH565-A did not show significant visible growth on petri dish culture (Fig. 5B). Cellobiohydrolase and xylanase activity of isolate STCH565-A was demonstrated by the formation of a clear visible zone around the colony on the solid media.

### General *Streptomyces* sp. STCH565-A genomic features

Table 2 summarizes the genomic features of *Streptomyces* sp. STCH565-A The total genome resulted in 8,492,054 bp (linear topology) with guanine-cytosine content (G+C) of 72.3% in 177 contigs after assembly. Genes encoding for plasmid replication machinery (ParA, ParB) were not found in the genome, suggesting the absence of plasmids. The functional annotation using the PATRIC RASTtk online server resulted in prediction of 7,493 coding-sequences (CDS) with 1,148 genes assigned to COG and 327 gene entries assigned to CAZy (Table 2), along with 67 tRNA and 2 rRNA (16S - 23S). The biosynthetic novelty index (BiNI) was estimated, obtaining a value of 587.

**Table 2:** Genome features of *Streptomyces* sp. STCH565-A. Assembly and annotation data, GenBank Bioproject number PRJNA845783.

**Feature**	***Streptomyces*** **sp.** **STCH565-A**
Genome topology	Linear
Chromosome size (bp)	8,492,054
GC content (%)	72.3
Protein-coding genes	7,493
rRNA genes	2
tRNA genes	67
Subsystems assigned by Patric	276
Genes assigned to COG	1148
Gene entries assigned to CAZy	327
Biosynthetic Novelty Index (BiNI)	587

### Carbohydrate-active enzyme exploration

To analyze the biotechnological potential of the *Streptomyces* sp. STCH565-A and thereby identify the proteins that could be involved in cellulose metabolism, the complete genome was analyzed using the dbCAN2 platform (https://bcb.unl.edu/dbCAN2/). All proteins containing catalytic modules (CD) belonging to carbohydrate active enzymes (CAZyme) and carbohydrate binding modules (CBM) were identified (Lombard et al., 2014). Further classification according to dbCAN2 showed that 167 proteins were classified as Carbohydrate-Active Enzymes, 68 of which are glycoside hydrolases organized in 24 distinct families.

Comparative analysis of *Streptomyces* sp. STCH565-A genome using the CAZy reference database (http://www.cazy.org/) allowed us to obtain a theoretical CAZyome, from the CAZy identified in the STCH565-A genome. Table 3 shows the information obtained, with emphasis on Glycosyl-Hydrolases and CBM.

**Table 3:** *Streptomyces* sp. *STCH 565A* proteins containing glycosyl hydrolase (GH) modules to lignocellulose deconstruction.

**CAZyme family**	**Gene ID**	**Enzyme activity (Cazy)**
*Glycosyl Hydrolases*		
GH1	565A_27_29 565A_37_46 565A_68_37 565A_86_23 565A_95_1	*β*-glucosidase; *β*-galactosidase; *β*-mannosidase
GH3	565A_20_18 565A_4_138 565A_146_1 565A_37_51 565A_3_167 565A_4_17 565A_34_27	*β*-glucosidase; xylan 1,4- *β*-xylosidase
GH5	565A_5_66 565A_4_137	Endo- *β*-1,4-glucanase/cellulase; Endo- *β*-1,4-xylanase; *β*-glucosidase
GH6	565A_35_84	Endoglucanase; Cellobiohydrolase
GH10	565A_24_87 565A_13_149	Endo-1,4- *β*-xylanase; Endo-1,3- *β*-xylanase
GH11	565A_65_11	Endo- *β*-1,4-xylanase; Exo-1,4- *β*-xylosidase
GH12	565A_40_7	Endoglucanase; Xyloglucan hydrolase
GH26	565A_2_225	*β*-mannanase; exo- *β*-1,4-mannobiohydrolase; *β*-1,3-xylanase
GH43	565A_14_77	*β*-xylosidase; *α*-L-arabinofuranosidase; Xylanase
GH51	565A_33_35	Endoglucanase; Endo- *β*-1,4-xylanase; *β*-xylosidase
*Auxiliary Activity Enzymes*		
AA1	565A_4_38	Laccase / p-diphenol:oxygen oxidoreductase / ferroxidase
AA3	565A_48_38 565A_51_44 565A_61_7	Cellobiose dehydrogenase; glucose 1-oxidase; Aryl alcohol oxidase
AA10	565A_68_3 565A_28_42 565A_36_19 565A_4_163	(Formerly CBM33) Lytic polysaccharide monooxygenases (LPMOs)
*Carbohydrate-Binding Modules*		
CBM2	565A_3_39	Cellulose-binding
CBM2 + AA10	565A_40_8	Cellulose-binding
CBM2 + GH6	565A_64_24	Cellulose-binding
CBM6	565A_64_2	Cellulose-binding
*Carbohydrate esterases*		
CE1	565A_18_8 565A_19_3 565A_37_43 565A_44_43 565A_4_171 565A_9_141	Acetyl xylan esterase
CE4	565A_13_78 565A_18_60 565A_20_70 565A_21_16 565A_21_17 565A_2_226 565A_36_26 565A_3_138 565A_5_79 565A_65_10 565A_6_147	Acetyl xylan esterase

The CAZyome of STCH565-A consisted of one or more domains of the CAZy family and includes Glycosyl-Hydrolases (GH), Glycosyl-Transferases (GT), Carbohydrate-Esterases (CE), and some redox enzymes that have auxiliary activities (AA) that function simultaneously, in addition to carbohydrate binding modules (CBM). Using the HMMER algorithm (Hidden Markov Model), 130 Glycosyl Hydrolases (GH), including CBMs and AA (copies included), were identified in the CAZyome of *Streptomyces* sp. STCH565-A GHs with EnG (E.C 3.2.1.4) enzymatic activity predicted in *Streptomyces* sp. STCH565-A are: GH10, GH5, GH26, GH51, GH12, CBM2+GH6, CBM6 and GH6. In total, 245 Cazy domains were identified, of which 130 were GH and CBM bound to them, plus five AA10 were predicted for *Streptomyces* sp. STCH565-A That represents 56% of all its Cazyome, dedicated exclusively to the hydrolysis of carbohydrates.

### Phylogenetic reconstruction of *Streptomyces* sp. STCH565-A

**Figure 6:**
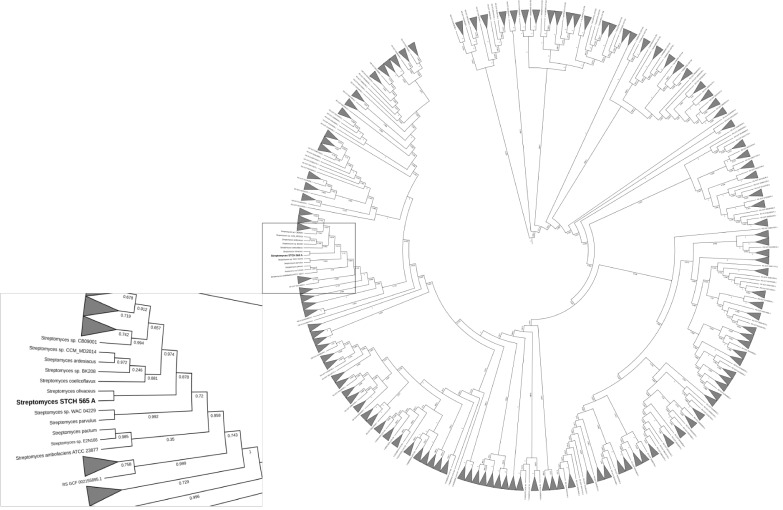
Phylogenetic reconstruction of *Streptomyces* sp. STCH565 A. Phylogenetic tree of 371 *Streptomyces* strains available. *Streptomyces* genomes selected from a set of 45,555 genomes using the GTDB-Tk workflow. The identification of 115 genes that served as markers were obtained and used to generate a multi-locus alignment. Clades were collapsed dat 2.5 average branch length and the resulting tree was visualized with the web-based tool Interactive Tree of Life.

Taxonomic annotation of contigs was generated using the Genome Taxonomy Database (GTDB-Tk) v1.0.2 with a set of 115 unique genes and markers predicted by Prodigal v2.6.3 and HMMER v3.1b1 respectively, to be finally concatenated and compared by GTDB-Tk as well as an Average Nucleotide Identity (ANI). After a taxonomic classification, the genome was more closely related to *Streptomyces olivaceus* (GCF_000721235.1) with a 97.91% FastANI comparison followed by *Streptomyces* sp. MH60 (GCF_002939385.1) with 90.27%, *Streptomyces rubrogriseus* (GCF_003112595.1) with 90.04% and *Streptomyces violaceorubidus* (GCF_000717995.1) with 89.99%. The visualization of the phylogenetic tree (Fig. 6) of *Streptomyces* STCH565-A also showed a close relationship with *Streptomyces coelicoflavus* (GCF_003112555.1) and *Streptomyces* sp. BK208 (GCF_004364245.1).

## Discussion

The Cuatro Ciénegas Basin (CCB) harbors diverse and unique microbiological diversity in its multiple ponds (Medina-Chávez et al., 2023; Moreno-Letelier et al., 2012; Rodriguez-Torres et al., 2017; Rodriguez-Verdugo et al., 2012; Taboada et al., 2018; Tapia-Torres et al., 2016; Velez et al., 2016). In particular, the Churince hydrological system is an unusual site of a marked oligotrophy but a great endemic microbial biodiversity (Cerritos et al., 2011; Elser et al., 2018; Minckley & Cole, 1968; Perroni et al., 2014; Taboada et al., 2018; Velez et al., 2016). Unique lineages of cultivable *Streptomyces* had previously been reported to thrive in the oligotrophic intermediate lagoon of Churince (Arocha-Garza et al., 2017), at least, before it completely dried up (Carson, 2022).

Our results show that 78% of evaluated CCB actinomycetes produce extracellular hydrolytic enzymes using carboxymethylcellulose (CMC) as the only carbon source. Few screening studies that find enzymatically “gifted microbes” able to hydrolyze cellulolytic substrates report the process behind their findings, we have compared our collection (Table S1) to other similar reports of bioprospection (Table S2). Compared to others, the CCB collection reveals a high potential for cellulolytic enzymes. CCB waters, particularly in the Churince, had shown unusual extreme stoichiometric nutrient ratios, generating nutritional stresses that dictate population dynamics perhaps more akin to earlier times in Earth’s history rather than more extant environments. Extreme oligotrophy has likely contributed to biological endemism (Souza et al., 2008; Souza et al., 2012a), which might explain the enriched community of highly cellulolytic actinomycetes, since this evaluated collection was selectively isolated for bioprospection without a carbon-usage bias.

A very recent study reveals the relationships between competition and polysaccharide decomposition, in which authors demonstrate that microbial diversity and competition affect the stability and function of cellulose-degrading communities where antagonism is prevalent in highly cellulolytic enrichment lines (Lewin et al., 2022). These results support our hypothesis that CCB is an enriched source of highly cellulolytic microorganisms that, because of oligotrophy and carbon limitation, harbor valuable microbial communities of gifted microbes.

Because nearly 80% of our collection was able to use CMC as only carbon source, we evaluated cellulose degradation in CCM1 agar medium in terms of enzymatic index (EI) to select the best possible cellulolytic candidate. Calculating EI is used to evaluate enzyme-producing prospects within the same genus (Teather & Wood, 1982) and was previously used for this purpose in other studies (Barzkar & Sohail, 2020; Fatokun, Nwodo & Okoh, 2016; Lewin et al., 2022; Sarkar & Suthindhiran, 2022). In this study, 12 out of 196 strains showed high EI values (≥ 3.0) and were considered as highly cellulolytic, so they were selected for further quantitative enzymatic assays (Fig. 4).

The volumetric enzymatic activity (Fig. 4) report shows us that the selected strain *Streptomyces* sp. STCH565-A presents higher values (0.27 U/mL) than the *Streptomyces* CC48 strain (0.11 U/mL) (Celaya-Herrera et al., 2020), previously isolated also from Cuatro Ciénegas (both values obtained when their optimization has not been carried out).

*Streptomyces* sp. STCH565-A volumetric activity values seem barely satisfactory when compared to other reports of non-optimized processes (Budihal, Agsar & Patil, 2016; Grigorevski de Lima et al., 2005). However, in this work, *Streptomyces* sp. STCH565-A- specific enzymatic activity measured for the crude extract (CE) of a non-optimized process was 149 U/mg after 15 days of non-fed submerged culture. Furthermore, specific enzymatic endoglucanase activity measured in crude extract, is also elevated after several days in the growth curve. After four days of culture, a specific enzymatic activity of 66.7 U/mg was found and after seven days, a specific enzymatic activity of 56.8 U/mg. Detailed studies of the characterization of the enzyme could give an approximation to an apparent affinity for the substrate. We can say that *Streptomyces* sp. STCH565-A has a high specific enzymatic activity when compared to another cellulolytic *Streptomyces*. For instance, a previous study reports 0.205 U/mg of cellulolytic activity (Fatokun, Nwodo & Okoh, 2016) while another measurement eaches 4.38 U/mg (Sujatha & Hemalatha, 2020).

The specific enzymatic activity is relevant in biotechnological terms (Budihal, Agsar & Patil, 2016) Because. Although there are different commercial cellulases obtained from fungal strains, their enzymatic activity is limited to volumetric activity, but not to its specificity to the substrate. This volumetric activity implies an increase in production costs because large amounts of these cellulases are required to achieve complete hydrolysis of polymeric matrices susceptible to enzymatic attack (Quinlan, Teter & Xu, 2010). Although high values of cellulolytic activity were reached under the evaluated conditions, the fermentation time necessary for *Streptomyces* sp. STCH565-A could be considered an obstacle for scaling processes because of the low bioconversion speed that leads to conversion: co-production of inhibitory compounds added to the size of the infrastructure (Manzanares, 2010). An alternative solution to consider as a research perspective would be the isolation and heterologous expression of the genes that were identified as responsible for the enzymatic activity (Arsov, Petrov & Petrova, 2021).

Regarding identification of selected strain, a previous work had already alerted the scientific community of the risks of under-appreciating diversity between strains with identical or nearly rRNA genes (Antony-Babu et al., 2017). Although this work phylogeny was based on the analysis of 115 marker genes, *Streptomyces* sp. STCH565-A was found to be closely related to *Streptomyces olivaceus*.

By and large, the degradation of polysaccharides into sugar monomers requires the synergistic action of several classes of carbohydrate-active enzymes (CAZyme). Among the CAZymes, glycoside hydrolases (GH) are the main family of enzymes involved in the degradation of polysaccharides such as starch and cellulose (Sidar et al., 2020).

Genomic analysis of the CAZyome showed that this *Streptomyces* sp. STCH565-A encodes the genes necessary for cellulose hydrolysis. The genes found in the CAZy database coincide with an important family of enzymes to degrade cellulose (Endoglucanase, GH12) as well as families of glycosyl-hydrolases such as GH5-GH6 (celobiohydrolases) and GH1-GH3 (β-glucosidases), forming a robust cellulolytic complex.

In the CAZy database, endoglucanases are classified into 15 different families, GH5, GH6, GH7, GH8, GH9, GH10, GH12, GH26, GH44, GH45, GH48, GH51, GH74, GH124 and GH148. Exoglucanases are divided into 5 families of GH5, GH6, GH7, GH9 and GH48 (Nguyen et al., 2018). Two of the most abundant domains found in the *Streptomyces* sp. STCH565-A genome belong to the GH1 and GH3 families (β-glucosidases [BG]), which are found in at least five gene copies; this is a considerable abundance compared to the CAZyome of the soil-isolated *Streptomyces yeochonensis* CN732 recently reported (Malik et al., 2020).

Endoglucanase variations in active site topology result in different substrate specificities. It was found that *GH6 PaCel6B* showed increased activity on CMC, which is a highly specific substrate for Endo-acting cellulases (Sidar et al., 2020). CMC is decrystallized cellulose and therefore contains more amorphous sites that are ideal for access to the cellulose chain by internally cleaving endoglucanases, whereas exoglucanase *GH6 PaCel6A* showed greater activity on insoluble microcrystalline Avicel than on CMC. Considering this and finding both GH12 and GH6 in the genome of our *Streptomyces* sp. STCH565-A, it is plausible that the CMC substrate could be catalytically attacked both in its amorphous structure and in the reducing and non-reducing ends, confirmed by the ability of our studied strain to grow on both substrates (Fig. 5C).

It was unexpected to find GH10 or GH11 enzymes in the *Streptomyces* sp. STCH565-A genome (Table 3), because both families had been identified mostly in host-associated *Streptomyces* and not within environmental strains (Book et al., 2016). What can be considered singular to our strain is to find the simultaneous presence of GH11 and 11 copies of acetyl-xylan-esterase in the genome (Table 3). This particular combination of enzymatic activity was previously found to be synergistic *in vitro,* improving the hydrolysis of pre-treated lignocellulosic biomass (Marasinghe et al., 2021); this experiment, however, was carried out using a cocktail of a commercially produced GH11 from *Streptomyces* and acetyl-xylan-esterase obtained from *Ochrovirga pacifica*. Identifying a bacterial strain harboring such a robust complex of CAZy domains makes *Streptomyces* sp STCH565-A an obvious candidate for future applications in the industry, either using the whole microorganism in a consortium or its genetic sources for heterologous expression and mixed enhanced cocktails.

*Streptomyces* sp. STCH565-A can use xylan as its only carbon source (Fig. 5C), which can be explained with the presence of several enzymes codified in the CAZyome: Glycosyl-Hydrolase GH10, GH11, GH26 and GH43 (xylanases), as well as multicopies of CE1 and CE4 (acetyl xylan esterases) (Table 3). These enzymes have been previously reported as auxiliary enzymes in xylan hydrolysis, whose activity eliminates recalcitrant lateral chains and exposes the xylan skeleton, improving the deconstruction process of cellulosic material (Vardakou et al., 2008).

The enzymatic attack is not limited to the catalytic machinery of glycosyl-hydrolases. CBM are ancillary modules attached to GH, and they are improving the catalytic functions of the CAZymes (Lombard et al., 2014) by gaining access to the insoluble substrate fraction of the cellulosic chain (Bernardes et al., 2019; Chalak et al., 2019). In this work, we report that additionally to the GH identified in the genome of *Streptomyces* sp. STCH565-A, a carbohydrate-binding module (CBM2) is codified in our strain, probably forming the CBM2-GH6 complex. CBMs do not have catalytic activity *per se*, but function as substrate-binding modules (Boraston Alisdair et al., 2004; Lombard et al., 2014). Studies suggest that the binding characteristic of a CBM enhances the catalytic function of CAZymes by directing the enzyme to the substrate and increasing substrate-enzyme proximity, as well as disrupting the crystallinity of the insoluble substrate fraction (Bernardes et al., 2019; Reyes-Ortiz et al., 2013); therefore, finding this CBM2-GH6 in the analyzed genome arouses great interest for this study. We could suggest that this module is one of the key characteristics to understand the affinity of the *Streptomyces* sp. STCH565-A strain for the evaluated substrate –CMC—which could be further confirmed by studies showing that removal of the CBM from the enzyme results in decreased enzyme activity and reduced enzyme stability (Cockburn et al., 2018).

Focused analysis on the auxiliary activities that bind to the cellulolytic complex to form a complete model for the enzymatic hydrolysis of cellulose, five copies of AA10 were found in the CAZyome of *Streptomyces* sp. STCH565-A These copies of AA10 suggest an important role in the results (Table 3). AA10 is a domain assigned by the CAZy database for lytic polysaccharide monooxygenases (LPMOs), which are copper-dependent enzymes that cleave polysaccharides through an oxidative mechanism. These enzymes have initially been used in synergy with glycoside hydrolases to enhance the saccharification of plant biomass and have been incorporated into state-of-the-art commercial enzyme cocktails for biofuel production (Johansen Katja, 2016; Moreau et al., 2019). In the STCH565-A genome, these LPMOs are present and naturally assume participation in cellulose degradation, without being added exogenously as occurs in other studies.

Overcoming recalcitrance of lignin hydrolysis is probably one of the urgent challenges in the deconstruction of hardwood and other lignocellulosic residues. *Streptomyces* sp. STCH565-A genome codifies auxiliary activities of AA1 (laccase) as well as CE1 (acetyl xylan esterase) and AA3 (aryl alcohol oxidase) whose activity has been shown to be essential in lignin hydrolysis (Ozer et al., 2020; Pothiraj, Kanmani & Balaji, 2006). Despite the presence of the lignin-related codified enzymes in its genome, *Streptomyces* sp. STCH565-A was not able to grow on lignin as a sole carbon source in the evaluated conditions. It is known that phenolic compounds derived from lignin inhibit lignocellulosic bioconversion (Qin et al., 2016), which might be alleviated by the concurrent action of the enzymatic activity of a community in an aquatic free-living context, such as the original environment from which *Streptomyces* sp. STCH565-A was retrieved. Enzymatic activity *in vitro* can further be experimented, optimizing growth media and conditions (Kumar & Chandra, 2020).

A broad study of the evolution of cellulolytic activity in the genus *Streptomyces* (Book et al., 2016) grouped highly cellulolytic organisms in two clades that related to host-associated origin. A third clade of less cellulolytic organisms grouped free-living *Streptomyces.* Authors demonstrated that highly cellulolytic (mostly host-associated) *Streptomyces* encompassed a particular combination of important enzyme families to deconstruct cellulose: (GH5, GH6, GH9, GH12, GH48, and AA10), and cellulose-targeting carbohydrate-binding module (CBM) families 2 and 3. However, a cellulolytic complex codified in the genome of our aquatic free-living *Streptomyces* sp. STCH565-A is also composed of cellulases (GH5, GH6, GH12, GH51), and a very robust complex of domains such as CBM2, CBM2+GH6, CBM2+AA10, AA10 and CBM6. It is notable that the codified CAZy inventory of STCH565-A seems closer to what is expected for a host-associated organism.

Previous findings show that free-living soil strains, which have evolved within diverse communities, have likely experienced limited selection for rapid cellulose-degrading activity in isolation (Book et al., 2016); our results, however, strongly contrast to high cellulolytic activity of our aquatic isolated strain *Streptomyces* sp. STCH565-A Considering stoichiometry imbalance and oligotrophy found in ponds of Cuatro Ciénegas (Souza & Eguiarte, 2018; Souza et al., 2018), it is likely that this selection pressure and high endemism, particularly in the Churince ponds whose water content had a deep ancient magmatic influence (Wolaver et al., 2012) might have exerted a selective pressure on the Actinomycete, and that highly cellulolytic *Streptomyces* sp. STCH565 A is probably related to its evolutionary origin.

## Conclusions

A systematic bioprospecting strategy to obtain highly cellulolytic actinomycetes was implemented in Cuatro Ciénegas Basing, producing the isolation and selection of an aquatic *Streptomyces* sp. STCH565-A, whose endoglucanse-specific activity stands out among other highly cellulolytic *Streptomyces.* Genomic analysis of this protruding strain reveals a robust CAZyome of singular simultaneous presence of valuable GH, AA and CBM domains, making our gifted strain a promising candidate for lignocellulosic bioconversion applications. Furthermore, we propose that this systematic approach could also be applied to other environmental contexts or to evaluate bacterial collections to retrieve highly cellulolytic microorganisms.

##  Supplemental Information

10.7717/peerj.16119/supp-1 Supplemental Information 1 Supplementary Material

## Additional Information and Declarations

### Field Study Permissions

The following information was supplied relating to field study approvals (i.e., approving body and any reference numbers): Access and sampling permission to the Natural Protected Area of Cuatro Cienegas was given by SEMARNAT.

### DNA Deposition

The following information was supplied regarding the deposition of DNA sequences: The whole genome project is available at GenBank: PRJNA845783.

### Data Availability

The following information was supplied regarding data availability: The genome assembly is available at Genbank: ASM2380537v1.
